# Association of Seasonal Hyperacute Panuveitis Syndrome with *S. pneumoniae* Endophthalmitis

**DOI:** 10.1016/j.xops.2026.101128

**Published:** 2026-02-21

**Authors:** Kenji Nakamichi, Anu Manandhar, Smita Shrestha, Miel Sundararajan, Manish P. Poudel, Biraj Man Karmacharya, Astaram Bade, Pradeep Banjara, Angira Shrestha, Angela Sandt, Gabrielle Turski, Ethan D. Buhr, Apoorva Chowdhary, Russell N. Van Gelder

**Affiliations:** 1 Department of Ophthalmology, University of Washington School of Medicine, Seattle, Washington; 2 Karalis Johnson Retina Center, University of Washington School of Medicine, Seattle, Washington; 3 Uveitis Division, Tilganga Institute of Ophthalmology, Kathmandu, Nepal; 4 Department of Ophthalmology, Dulikhel Hospital, Dulikhel, Nepal; 5 Department of Laboratory Medicine and Pathology, University of Washington, Seattle, Washington; 6 Department of Neurobiology & Biophysics, University of Washington, Seattle, Washington

**Keywords:** Seasonal hyperacute Panuveitis Syndrome, SHAPU, Metagenomic sequencing, Streptococcus pneumoniae, Endophthalmitis

## Abstract

**Purpose:**

To identify potential infectious agents in cases of seasonal hyperacute panuveitis syndrome (SHAPU) from vitreous biopsies of patients with this disorder.

**Design:**

A retrospective cohort analysis.

**Subjects:**

Vitreous biopsies were obtained during the course of care from 53 subjects with SHAPU.

**Methods:**

DNA extraction and whole genome shotgun sequencing was performed using Oxford Nanopore long read sequencing. Sequences were matched against microbial and human databases. Visual outcomes at presentation and at 6 months were recorded.

**Main Outcome Measures:**

Identification and characterization of metagenomic sequences in vitreous isolates from subjects with SHAPU.

**Results:**

Adequate DNA for sequencing was obtained from 32 SHAPU subjects. Fifteen samples yielded bacteria on culture, with 14 *S. pneumoniae* and 1 *S*. *aureus* isolate recovered. Bacterial DNA was detected by whole genome sequencing in 29 of 32 cases. *S*. *pneumoniae* was the predominant organism recovered. Bacterial genomic loads ranged up to 10 000 bacteria/human cell, indicating active infection. No pathogens were detected in control samples. Reconstruction of bacterial genome was possible in 7 SHAPU cases and indicated diverse *S. pneumoniae* subtypes associated with individual cases. Sufficient DNA remained for analysis of torque teno virus by qualitative polymerase chain reaction in 17 cases, of which 13 were positive. Visual outcomes were mixed, with 7 patients having hypotonous eyes at 6 months, but 8 patients having better than 20/200 vision. No relationship could be discerned between presenting bacterial load and visual outcome.

**Conclusions:**

The majority of SHAPU cases show molecular evidence for concurrent *S. pneumoniae* infection. Good visual results are possible in treating SHAPU as endophthalmitis.

**Financial Disclosure(s):**

The authors have no proprietary or commercial interest in any materials discussed in this article.

Seasonal hyperacute panuveitis syndrome (SHAPU) was first described by Malla in 1975, in a report of 10 cases of sudden unilateral blinding endophthalmitis in Nepalese children.^1^ The condition was named SHAPU by Upadhyay et al^2^ in 1984. Malla named it seasonal endophthalmitis and described it as a noninfective suppurative inflammation of the inner eye in children.^3^ Seasonal hyperacute panuveitis syndrome predominantly occurs in Nepal immediately after the monsoon season (August to September) until the peak of winter (December–January), primarily during odd years.^4^, ^5^, ^6^ More recently, sporadic summer outbreaks with atypical presentations during even years have also been noted.^6^^,^^7^ In 2020, the first possible cases of SHAPU outside of Nepal were reported in Bhutan.^8^ Patients are usually children, presenting with sudden-onset unilateral redness, photophobia, and white pupillary reflex.^4^^,^^7^^,^^9^ Hypopyon and dense fibrinoid reaction in the anterior chamber are often observed, with shallowing of the anterior chamber and ocular hypotension in the late stages. Patients experience rapidly progressive vision loss, and in two-thirds of cases, present with a blind eye.^5^ Therapeutic regimens remain largely ineffective if not utilized early in the disease course, although treatment with vitrectomy and intravitreal antibiotics have shown efficacy in some small series.^4^^,^^5^^,^^10^

Exposure to moths has been associated with SHAPU, with many patients reporting direct contact with moths prior to onset of symptoms.^4^^,^^9^^,^^11^, ^12^, ^13^ Studies have reported Tussock moths, as well as *Gazalina* moths (a group of processionary moths) as possible agents given reports of contact from patient history and emergence patterns chronologically associated with SHAPU outbreaks.^1^, ^2^, ^3^^,^^11^^,^^14^ It has been hypothesized that SHAPU pathogenesis is driven by introduction of microbes or toxins via ocular microtrauma by moth setae or “hairs,” given their identification in the ocular structures of some patients.^11^^,^^14^ Various organisms, including anelloviruses and bacteria (*S. pneumoniae, S*. *aureus,* and *Acinetobacter)* have been isolated from vitreous fluid samples of SHAPU patients, with at least 14% of SHAPU cases shown to be bacterial endophthalmitis by bacterial culture in previous studies.^14^^,^^15^ Although these findings suggest an infectious cause, an immunologic response to an antigen on moth hair causing uveal inflammation cannot be ruled out.

The illness burden of SHAPU in Nepal remains large, with the most recent outbreak affecting more than 1500 individuals residing in the Gandaki Province.^16^ Identification of infectious pathophysiology could lead to an optimal protocol for the management of SHAPU cases. In this study, we sought to further identify potential infectious etiologies of SHAPU by metagenomic sequencing of vitreous samples from SHAPU patients.

## Methods

This study was approved by the Ethics Panel of Nepal Health Research Council and complies with the Declaration of Helsinki. All patients gave informed consent or, in the case of minors, assent with parental consent. Patients presenting with signs and symptoms consistent with SHAPU were recruited from 2017 to 2019, principally from the Pokhara region of Nepal. Treatment of subjects was at the discretion of the attending physician and generally included vitreous tap and injection of antibiotics for patients with acuity better than light perception (LP) and pars plana vitrectomy with intravitreal antibiotics for patients with vision of LP or worse.

Samples obtained in the course of routine care (i.e., vitreous biopsy) were subjected to standard laboratory culture at Tilganga Institute of Ophthalmology. Remaining vitreous was frozen at –80°C and shipped on dry ice to the Department of Ophthalmology at the University of Washington. DNA was extracted using the Qiagen DNeasy Blood and Tissue DNA Kit (Qiagen). DNA concentration was measured using Invitrogen double-stranded DNA high-sensitivity fluorescent assay (Thermo Fisher) with Qubit fluorimeter, which has a manufacturer-reported lower limit of detection of approximately 5 pg/μL. Samples with less than 1 ng/ul DNA were not processed further. Samples were linearly amplified using Phi29 polymerase using Qiagen RepliG ultrafast kit (Qiagen) per the manufacturer's recommendations. Shotgun whole genomic sequencing was performed using polymerase chain reaction (PCR)-free SQK-LSK109 ligation sequencing kit on the Oxford Nanopore Minion using R9.4.1 chemistry (Oxford Nanopore) platform using the manufacturer's protocol, utilizing 1 flow-cell per sample. Samples were run until less than 100 active pores were functioning or 72 hours elapsed, whichever occurred first. Base calling was performed using Guppy (Oxford Nanopore). Negative (“Blank”) samples beginning with sterile water were run with each batch of DNA extraction. Sequences were analyzed using the Scalable Metagenomics Analysis Research Tool^17^ using the GenBank Release 209 dataset. Bacterial sequences indigenous to the “kitome,” including *Ralstonia*, *Burkholderia*, and *Bradyrhizobium* species^18^ were detected in all samples including negative controls and were redacted from results. Clustering analysis was performed using Jaccard similarity matrices.^19^ De novo assembly of samples with greater than 15 times coverage by reference alignment was completed using Flye.^20^ The scaffolds were aligned to the reference genome, and multilocus sequence typing (MLST) was done using fastMLST.^21^ Comparison of sequences with reference *S. pneumoniae* utilized RefSeq assembly GCF_001457635.1 (strain SNTC7465) and variants and pairwise reference comparisons were completed using publicly available alignment methods (minimap2 -> pepper-margin-deepvariant/clair3/samtools + bcftools) as described.^22^ Molecular serotyping of *Streptococcus pneumoniae* was performed with PneumoKITy^23^ and PfaSTer.^24^ Polymerase chain reaction and quantitative PCR for human actin, 16S bacterial DNA, and torque teno virus (TTV) were performed as described.^18^ Quantitative PCR for *S. pneumoniae* was performed using *S. pneumoniae*-specific primers^25^ along with a quantified clone amplicon control. Statistical analysis was performed using Prism GraphPad 9.5.

## Results

Samples from a total of 53 SHAPU subjects were obtained. Additionally, vitreous from 7 subjects undergoing pars plana vitrectomy for noninfectious causes underwent DNA extraction. Thirty-two of the SHAPU-derived samples yielded sufficient DNA for further analysis (>1 ng/μl) and are reported here. Demographics of subjects with sufficient DNA for analysis are shown in Table 1. Demographic and initial treatment data were not available for 3 subjects. For the remaining 29, ages ranged from 1 to 41, with a mean of 10.1 years. There were 11 males and 18 females. Presenting visual acuity ranged from 20/80 to no light perception (NLP). Thirteen patients underwent tap and injection for treatment, while 16 underwent initial pars plana vitrectomy at attending physician’s discretion. Fifteen of the 32 vitreous samples yielded bacteria on standard culture, with 14 of these positive for *S. pneumoniae* and 1 positive for *S*. *aureus.*

**Table 1:** Subject Demographics

Subject ID	Age (Yrs)	Gender	Laterality (OD/OS)	Vision at Presentation	Intervention	Vision at 6 Months	Globe Status
1	19	F	OS	20/80	T&I	F&F	Preserved
2	4	F	OD	F&F	T&I	20/20	Preserved
3	1	F	OD	-	PPV	DNFFL	Phthisis
4	7	F	OD	LP	PPV	LP	Hypotony
5	–	–	–	–	–	–	–
6	–	–	–	–	–	–	–
7	6	F	OS	LP	PPV	20/200	Preserved
8	8	F	OD	LP	PPV	–	–
9	1	F	OD	–	PPV	DNFFL	Hypotony
10	7	M	OD	LP	PPV	20/40	Preserved
11	13	F	OS	LP	PPV	20/30	Preserved
12	24	F	OS	LP	PPV	LP	Hypotony
13	13	M	OS	LP	PPV	20/120	Preserved
14	6	M	OD	LP	T&I	20/20	Preserved
15	4	M	OS	–	PPV	DNFFL	Phthisis
16	0.75	M	OD	–	PPV	–	–
17	3	M	OS	–	PPV	–	–
18	9	F	OD	–	PPV	DNFFL	Hypotony
19	3	F	OD	–	T&I	NLP	Phthisis
20	1.4	M	OS	F&F	T&I	F&F	Preserved
21	1	F	OD	–	T&I	HM	Preserved
22	23	F	OD	LP	T&I	NLP	Phthisis
23	–	–	–	–	–	–	–
24	16	M	OD	LP	PPV	–	–
25	24	M	OD	20/200	T&I	20/30	Preserved
26	0.83	F	OD	–	PPV	NLP	Preserved
27	4	F	OD	20/400	PPV	20/400	Preserved
28	9	F	OD	NLP	T&I	–	–
29	28	M	OS	20/300	T&I	20/20	Preserved
30	12	F	OD	LP	T&I	–	–
31	5	F	OD	HM	T&I	20/30	Preserved
32	41	M	OD	HM	T&I	20/30	Preserved

DNFFL = does not fix & follow light; F&F = fix and follow; HM = hand motion; LP = light perception; NLP = no light perception; OD = right eye; OS = left eye; PPV = pars plana vitrectomy; T&I = tap and inject.

DNA yields from SHAPU patients averaged 50 ng/μl, compared with <2 ng/μl for control noninfectious vitrectomy samples and <1 ng/μl for blank controls, reflecting increased cellularity in inflamed tissue. Whole genome sequencing (WGS) yielded between 7477 and 190 663 370 human reads per sample with mean of 3 042 727 reads (Fig S1 and Table S1, available at www.ophthalmologyscience.org). Digital karyotyping^26^ showed expected yield of human sequences per chromosome in all samples, indicating high-quality DNA and linear whole genome amplification.

**Figure 1:**
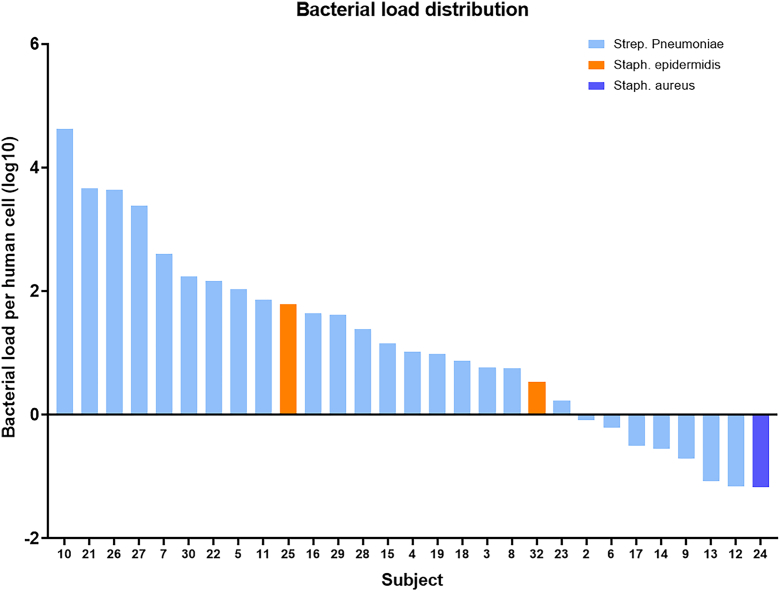
Bacterial load per subject. Total bacterial sequence reads were normalized to total human reads, assuming *S. pneumoniae* genome size of 2 MB and human diploid genome size of 6.6 GB/cell.

Bacterial sequences were recovered on long-read metagenomic sequencing in 29 of 32 samples, including 15 of 17 samples that were negative by culture (Table 2). None of 7 control vitreous samples from uninflamed subjects demonstrated detectable bacteria. All SHAPU biopsies positive by culture for *S. pneumoniae* revealed *S. pneumoniae* as the predominant sequence in WGS. One sample was positive for both *S*. *epidermidis* and *S*. *aureus* sequences. One sample revealed TTV on sequencing but no bacteria. In samples positive for *S. pneumoniae,* the number of bacterial reads recovered varied from 17 to 686 195. Quantification of bacterial reads per human cell, normalizing for genome sizes of *S. pneumoniae* and human, revealed infectious loads ranging from >10 000 bacteria per human cell to 1 bacterium per 100 human cells (Fig 1). Analysis of quantitative PCR for *S. pneumoniae* determined that 17 of the 32 samples had >10 000 bacterial genomes/mL. Mean *S. pneumoniae* bacterial load per human cell (log_10_) was 1.67 in the culture-negative group compared with 3.62 in the culture-positive subjects (*P* = 0.012, Mann–Whitney *U* test). However, 4 of the 12 culture-negative, *S. pneumoniae* sequence-positive samples yielded >1 bacterium/human cell.

**Table 2:** Culture and Whole Genome Sequencing Results

Subject ID	Organism Cultured by Conventional Microbiology	Organism per Whole Genome Sequencing	Bacterial Load/Human Cell (Log 10)
1	Negative	Negative	0
2	Negative	*S. pneumoniae*	–0.0924
3	*S. pneumoniae*	*S. pneumoniae*	0.7701
4	*S. pneumoniae*	*S. pneumoniae*	1.1725
5	Negative	*S. pneumoniae*	2.0337
6	Negative	*S. pneumoniae*	–0.2164
7	Negative	*S. pneumoniae*	2.6042
8	*S. pneumoniae*	*S. pneumoniae*	0.7572
9	Negative	*S. pneumoniae*	–0.7021
10	*S. pneumoniae*	*S. pneumoniae*	4.6237
11	*S. pneumoniae*	*S. pneumoniae*	1.8591
12	Negative	*S. pneumoniae*	–1.1649
13	Negative	*S. pneumoniae*	–1.0790
14	Negative	*S. pneumoniae*	–0.5486
15	*S. pneumoniae*	*S. pneumoniae*	1.1507
16	*S. pneumoniae*	*S. pneumoniae*	1.6435
17	Negative	*S. pneumoniae*	–0.4973
18	Negative	*S. pneumoniae*	0.8729
19	*S. pneumoniae*	*S. pneumoniae*	0.9871
20	Negative	Negative	0
21	*S. pneumoniae*	*S. pneumoniae*	3.6669
22	*S. pneumoniae*	*S. pneumoniae*	2.1645
23	Negative	*S. pneumoniae*	0.2338
24	*Staphylococcus aureus*	*S. aureus*	–1.1691
25	*S. pneumoniae*	*S. epidermidis/S. aureus*	1.7917
26	*S. pneumoniae*	*S. pneumoniae*	3.6366
27	*S. pneumoniae*	*S. pneumoniae*	3.3830
28	Negative	*S. pneumoniae*	1.3893
29	Negative	*S. pneumoniae*	1.6210
30	*S. pneumoniae*	*S. pneumoniae*	2.2429
31	Negative	Torque teno virus	n/a
32	Negative	*S. epidermidis*	0.5300

Seven samples yielded greater than 85% sequence breadth relative to reference *S. pneumoniae* genome and were sufficient to cluster against existing *S. pneumoniae* clades (Fig S2, available at www.ophthalmologyscience.org, top panel) by Jaccard sequence analysis. Comparing these sequences with high coverage breadth to the 139 full-length *S. pneumoniae* sequences found in NCBI GenBank, we found the 7 samples do not form a clear cluster within the known sequences and show broad genotypic distribution (Fig S2, middle panel). This suggests that these sequences do not reflect carry-over or cross-contamination and that SHAPU is not associated with a specific variant strain of *S. pneumoniae*. Multilocus sequence typing was performed with fastMLST^21^ using the 7 loci-defining MLST type. Of these, only sample s19_v26 yielded a positive identification, identified as MLST Type 9. The remaining samples did not yield a high confidence MLST match. However, clustering of single-nucleotide polymorphisms among the 7 genes used in MLST typing (spi, recP, xpt, ddl, aroE, gdh, and gki) demonstrated a broad distribution within known MLST clades, again suggesting that the recovered *S. pneumoniae* sequences do not appear to belong to a single or small number of strains (Fig S2, bottom panel). Finally, the 7 high coverage isolates were analyzed for serotype using molecular analysis tools PneumoKITy^23^ and PfaSTer.^24^ Only sample s19_v22 yielded a high confidence match by both methods, corresponding to serotype 19A (“pass” by PneumoKITy and 99% confidence by PfaSTer). Remaining samples were less than 50% probability for match by PfaSTer (Fig S2, lower panel).

**Figure 2:**
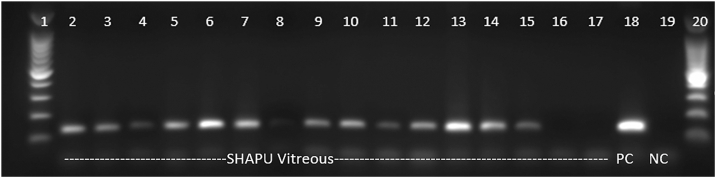
Qualitative PCR for torque teno virus in 17 samples with adequate DNA for analysis. NC = negative control; PC = positive control; PCR = polymerase chain reaction; SHAPU = seasonal hyperacute panuveitis syndrome.

Previous work has suggested that TTV can be found in most cases of SHAPU.^15^ Torque teno virus has also been found in many cases of bacterial endophthalmitis, where its presence is a negative prognostic indicator.^27^^,^^28^ As the TTV genome is exceedingly small (3.7 kb), it is difficult to detect in WGS. We therefore performed qualitative PCR for TTV on 17 samples with sufficient DNA remaining (Fig 2). Thirteen of 17 were positive for TTV, comparable to the ∼90% positivity reported previously.^15^

**Figure 3:**
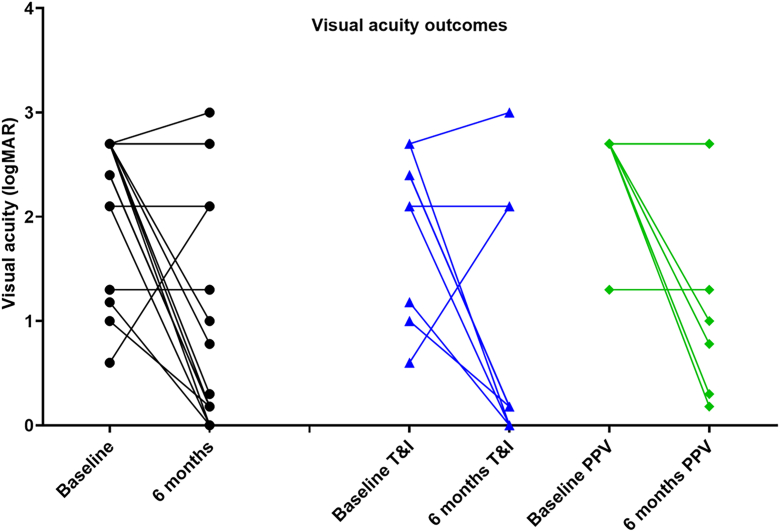
Six-month visual acuity outcome, stratified by T&I and PPV. NLP = logMAR 3 in this analysis. logMAR = logarithm of the minimum angle of resolution; NLP = no light perception; PPV = pars plana vitrectomy; T&I = tap and inject.

**Figure 4:**
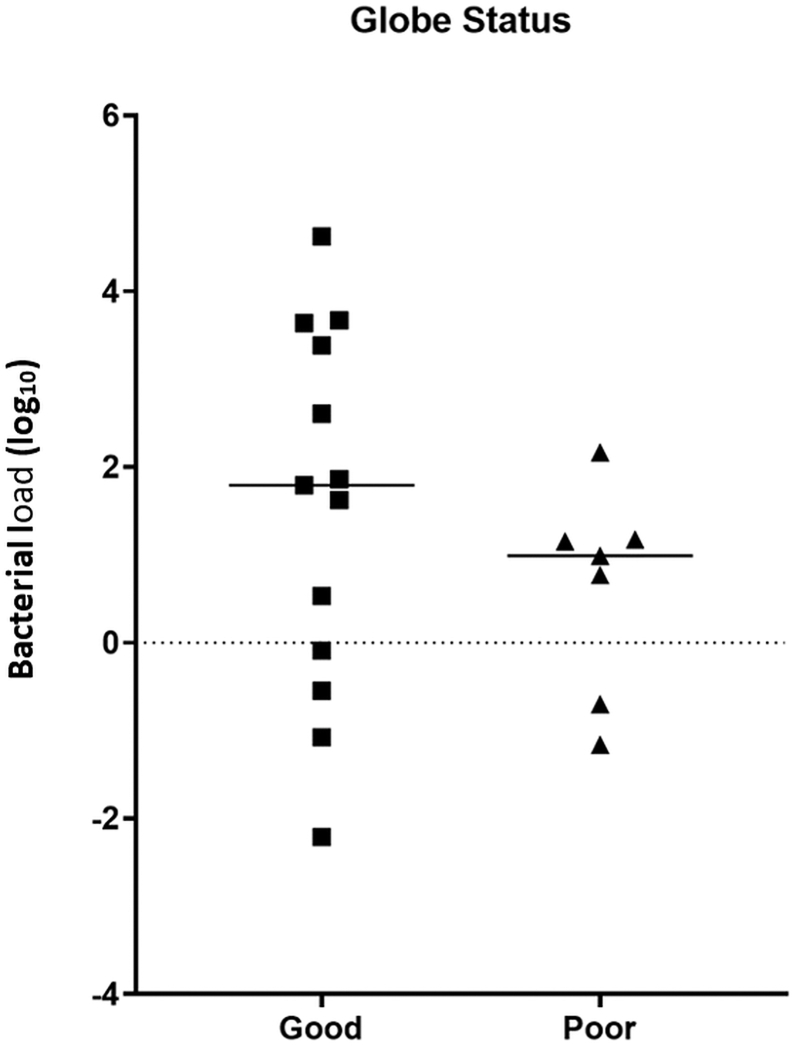
Globe status (intact = good; hypotonous/phthisical = poor) as a function of baseline bacterial load.

Six-month outcomes were available for 23 of the subjects in the study, and visual acuity data were available for 17 of these. Of these 17 patients, 9 underwent vitreous tap and injection (T&I), and 8 underwent pars plana vitrectomy (PPV). As shown in Figure 3, most patients demonstrated an improvement in vision from initial presentation, with 6 of 9 patients receiving tap and injection achieving better than 20/200 vision, and 3 of 8 patients receiving vitrectomy achieving vision better than 20/200. It is important to note that acuities tended to be worse at presentation in patients who underwent vitrectomy. Eight patients were hypotonous or phthisical at the 6-month visit (6 in the vitrectomy group and 2 in the tap-and-inject cohort). Initial acuities were only available for 3 of the eyes that became hypotonous or phthisical; in all cases, these were LP or NLP at presentation. We tested whether bacterial load was predictive of hypotony or phthisis but found no relationship between load at presentation and globe status at 6 months (Fig 4).

## Discussion

Seasonal hyperacute panuveitis syndrome is an idiopathic severe panuveitis affecting predominantly children in Nepal.^4^ It is characterized by rapid, progressive inflammatory exudates in the anterior chamber and vitreous with hypotony and profound vision loss.^5^^,^^9^ The disease is typically unilateral and occurs in immunocompetent patients. It is also seasonal in nature, coinciding with the end of the monsoons, and has been strongly linked to interaction with moths, particularly of the *Gazalina* genus. Moth “hairs” have been found embedded in eyes with SHAPU.^3^^,^^11^^,^^12^^,^^13^ However, whether the disease represents an innate response to moth antigen or introduced infection has not been definitively demonstrated. In addition, some posit that there is a subtype of SHAPU, which is not moth-associated, particularly given variability in the seasonal pattern in more recent years.^5^

Previous studies have shown variable microbiologic yield for causative organisms. A majority of reported SHAPU cases have been culture- and (when tested) PCR-negative for bacteria. In those with a positive result, the pathogens isolated have been varied, ranging from varicella zoster virus, *S. pneumoniae, Acinetobacter,* and anelloviruses such as TTV.^5^^,^^14^^,^^15^ Given the lack of consensus on etiology of the inflammation (particularly the question of infectious versus innate immune pathogenesis), treatment course has differed among reports. A combination of both systemic and local (topical, subconjunctival, and intravitreal) antibiotics and steroids has been reported,^29^ as has surgical intervention in the form of pars plana vitrectomy.^10^

Whole genome sequencing offers an opportunity to identify organisms of interest with a greater degree of sensitivity than conventional culture for detection of causative organisms in endophthalmitis.^30^ Unlike 16S-based pan-bacterial PCR diagnostics, WGS is agnostic to etiology, and can detect any DNA-based life form, including bacteria, fungi, parasites, and DNA viruses. Approximately 50% of samples from this cohort of SHAPU patients were culture-positive for *S. pneumoniae,* while ∼90% of vitreous samples from these patients had molecular evidence on WGS for *S. pneumoniae*. In many cases, the bacterial multiplicity of infection was large, on the order of >100 bacteria/human cell, with most cases showing multiplicity of infection >1. Sequence analysis of full-length reconstructions of recovered *S. pneumoniae* genomes suggests that no specific strain of *S. pneumoniae* is associated with SHAPU. Of note, 3 cases did not have detectable bacterial DNA. Whether this represents failure to detect bacterial DNA, or an innate mechanism of disease in these cases is not clear. It is possible as well that presence of moth “hairs” is inflammatory even in the absence of infection; such a mechanism is thought to be responsible for “tarantula hair” panuveitis.^31^^,^^32^

Seven samples yielded sufficient sequence for near-complete genome reconstruction. Of note, only 2 samples could be definitively linked to MLST type or serotype (one each). While it is theoretically possible that the remaining samples represent new strains or serotypes, it is more likely that inability to perform accurate typing is due to insufficient sequence for the exact nucleotide polymorphism matching required for the computation tools for MLST (fastMLST^21^) or serotype (PneumoKITy^23^ and PfaSTer^24^) determination. These tools were all developed for use with short-read sequencing platforms; long-read tools such as Nanopore tend to score discrepancies with reference genome (such as small indels) differently, which may erode confidence.^33^ Previous attempts to use Oxford Nanopore sequence data for *S. pneumoniae* MLST typing have shown poor exact agreement (∼16%) with the identification of same samples using Illumina sequencing.^34^ Nonetheless, with respect to strain typing, it is clear that the *S. pneumoniae* sequences recovered did not belong to a single cluster or clade, suggesting that SHAPU-associated *S. pneumoniae* will not be due to a very specific isolate. Future studies should include performance of direct sequencing on positive microbial cultures as well as hybrid long-read and short-read approaches or targeted PCR amplification of MLST and serology-determining genes.

Consistent with previous studies,^15^ we found TTV DNA in >80% of SHAPU samples by PCR. Torque teno virus has been found in ∼50% of postoperative endophthalmitis cases and is thought to be a marker of severe inflammation rather than a causative agent.^27^ The presence of TTV is a strong negative prognostic indicator for the need for eyes to undergo additional surgery.^28^ The finding of TTV in these cases is consistent with the severe inflammation in these eyes.

Outcomes in this series were mixed, with 8 of 23 subjects with 6-month data available having phthisical or hypotonous eyes. Of the remainder, 8 had 6-month acuity equal or better than 20/40, suggesting that good visual outcomes are possible with this condition, but such outcomes are in the minority. Patients with favorable outcomes included 2 patients undergoing vitrectomy, and 6 undergoing tap and injection procedures. We did not see a correlation between bacterial load and outcome in this study. However, patients presented at different times in their course, and it is possible that nonviable bacteria contributed to DNA quantification in some patients.

Overall, the results of this study suggest that SHAPU is frequently associated with bacterial endophthalmitis, with the majority of samples testing positive for *S. pneumoniae* by WGS. As the 7 samples for which full-length genomes could be reconstructed appeared derived from differing clades, it is unlikely that these results are artefactual. These results suggest that aggressive antimicrobial therapy, comparable to that employed for endophthalmitis, may be appropriate for patients with this condition. Large randomized controlled trials have been limited to postsurgical endophthalmitis, but they suggest that eyes with a visual acuity of LP or worse should be treated with pars plana vitrectomy along with intravitreal antibiotics.^35^ (However, it should be noted that this study is not necessarily applicable to endogenous endophthalmitis, and there is question as to whether these guidelines should be applied in non-postsurgical cases^36^). *S. pneumoniae* has been shown to be an aggressive pathogen in postoperative endophthalmitis, with 1 group reporting NLP vision in 10 of 27 patients despite appropriate antibiotic therapy,^37^ and a more recent study reporting LP or NLP vision in 32 of 38 eyes.^38^ Byanju et al^10^ reported on the benefits of pars plana vitrectomy in SHAPU, in particular, in conjunction with subconjunctival and topical steroid (dexamethasone) and antibiotic (gentamicin and chloramphenicol, respectively). In this study, of 18 patients, 14 underwent pars plana vitrectomy, and 50% of these achieved vision of 20/200 or better. Two of the 4 patients who underwent medical therapy achieved 20/200 or better; however, it is important to note that medical therapy in these cases consisted of systemic, posterior sub-Tenon's, and topical steroids; antibiotics (chloramphenicol) were administered as hourly topical drops only.

While metagenomic sequencing was valuable in identifying potential pathogens underlying SHAPU, limitations in current methodology do not allow as complete analysis as would be desirable. The majority of samples did not yield sufficient fraction of the genome to allow characterization of the *S. pneumoniae* capsule sequences, for instance, which are known to be determinants of pathogenicity.^39^ Further, detection of potential bacterial antibiotic resistance genes in pneumococcal DNA sequence does not necessarily correlate with observed resistance (in some cases due to low expression of these genes^40^), which still requires traditional culture and sensitivity testing. Finally, the Scalable Metagenomics Analysis Research Tool dataset was generated based on release 209 of GenBank,^17^ which was generated in 2015. Most common pathogens are present in this dataset, but it is conceivable that more recently discovered pathogens might not be detected using this dataset.

The finding that most cases of SHAPU in this series had evidence of *S. pneumoniae* infection raises the question of utility of *S. pneumoniae* vaccination. Nepal’s Ministry of Health has embedded a 10-valent *S. pneumoniae* vaccine in the required immunization schedule for infants at age 6 weeks, 10 weeks, and 9 months in 2015.^41^ Per World Health Organization data, by 2017, 80% of Nepalese infants had completed immunization. As the present study enrolled individuals presenting in 2017 to 2019, only 6 of the 32 subjects would have been young enough to have been included. It would be of substantial interest to determine if these children had received vaccine. It would also be of interest to determine the serotype of the *S. pneumoniae* associated with all SHAPU cases, as the 10-valent vaccine does not cover several emerging variants, including serotypes 3, 6A, and 19A.^42^ Of note, the one sample in the current study with definitive serotyping was associated with serotype 19A, which is not included in the PCV10 vaccine currently in use in Nepal.

These results provide support for the hypothesis that *S. pneumoniae* infection introduced into the eye via moth setae may underlie the pathophysiology of SHAPU. Humans are generally considered the primary host for *S. pneumoniae,* and the bacteria has been detected on the conjunctival surface of normal individuals, particularly children. In one study of conjunctival flora in 6440 children in China, 301 grew *S. pneumoniae* (making it the third most commonly cultured bacterium behind *Corynebacteria* and *Staphylococcus epidermidis*).^43^ However, if the moth setae are creating a general conduit for introduction of conjunctival fauna intraocularly, it is unclear why such a preponderance of cases are positive for *S. pneumoniae* and not *S. epidermidis* (which is also capable of causing bacterial endophthalmitis, although 3 *Staph-*positive cases were detected in the present study).

An interesting similar conundrum exists for the horse disease equine amnionitis and fetal loss, also known as mare reproductive loss syndrome.^44^ In this disease (that also has annual epidemics occurring in certain years and locations), pregnant horses grazing in proximity to eastern tent caterpillars in the United States or oak processionary caterpillars in Australia experience high rates of fetal loss. Pathology studies have shown caterpillar setae breach gastrointestinal mucosal integrity, which may lead to bacteremia. Feeding pregnant mares with shed caterpillar exoskeletons induced this condition, and mares were found to have exoskeleton setae in the allantochorion in most animals.^45^ Aborted fetuses were culture-positive for non-beta hemolytic *Streptococci* in >50% of cases.^46^ For SHAPU, one speculative hypothesis is that the moth setae may preferentially carry *S. pneumoniae*; however, while bacteria belonging to the genus *Streptococcus* have been occasionally cultured or detected in other insects such as houseflies^47^ and have been found by 16S metagenomics in the gut flora of the unrelated adult *Plutella xylostella* moth,^49^ and while *S.* *pneumoniae* have rarely been cultured from cockroaches in hospital settings,^48^ there are no data to date to suggest that Tussock or *Gazalina* moths may serve as a reservoir for *S*. *pneumoniae* nor that their setae harbor these bacteria. Further analysis of these moths and their setae is necessary to evaluate this possibility.

In summary, the results of the present study suggest that the majority of SHAPU cases have significant levels of detectable *S. pneumoniae* DNA in vitreous. Management of these cases as presumptive endophthalmitis may be warranted.
