# Supplementary material for: Association of Seasonal Hyperacute Panuveitis Syndrome with S. pneumoniae Endophthalmitis
Source: Ophthalmol Sci. 2026 Feb 21;6(5):101128. doi: 10.1016/j.xops.2026.101128 (PMC13059302; doi:10.1016/j.xops.2026.101128)
Supplement: Figures S1 and S2 and Table S1 [file mmc1.pdf]

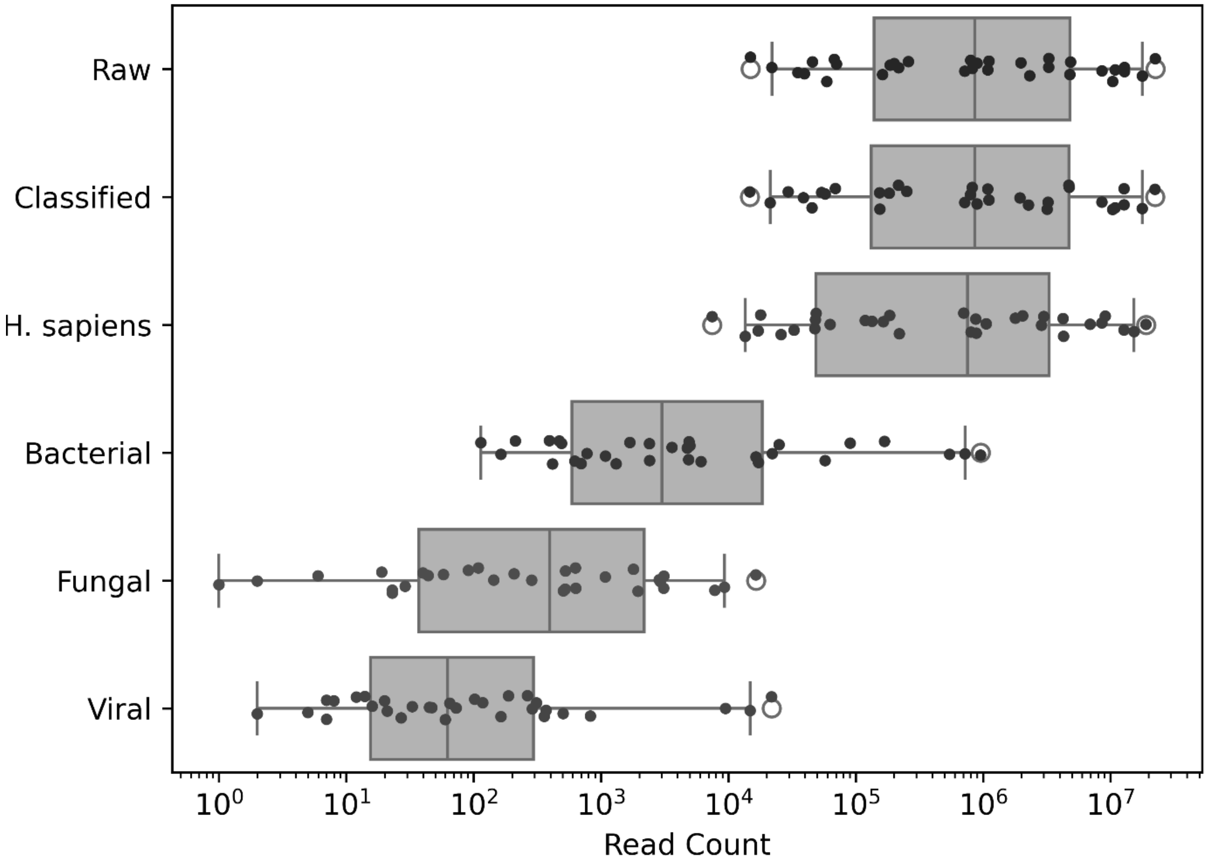

Figure S1. Raw read count distribution, represented as box and whisker with grey region representing 25th to 75th percentile of distribution and center line the median. Open circles represent maxima and minima; sequence sources without a lower open circle had samples with zero detectable sequences.

Type	count	mean	std	min	25%	50%	75%	max
Raw	32	3,867,624	5,879,858	14,940	139,797	862,890	4,835,010	22,578,077
Classified	32	3,838,033	5,854,124	14,749	133,072	861,807	4,737,962	22,446,634
Homo sapiens	32	2,991,790	4,915,969	7,477	48,849	756,609	3,320,208	19,066,370
Bacterial	32	83,438	224,406	0	591	3,014	18,501	958,905
Fungal	32	1,792	3,464	0	37	397	2,183	16,468
Viral	32	1,569	4,795	0	16	63	295	21,877

Table S1. Read count summary.

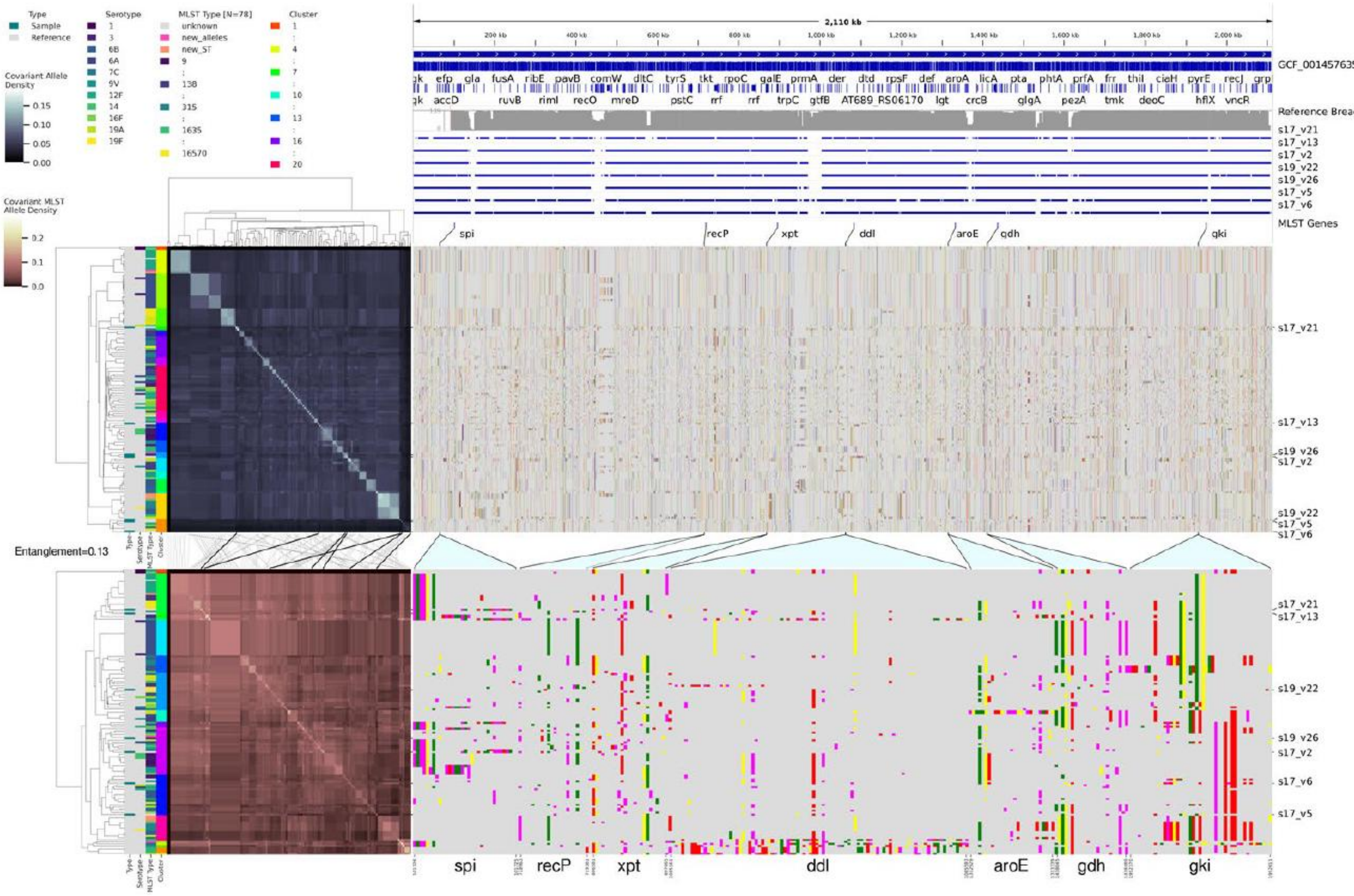

Figure S2: (top) shows the integrated genomics viewer panel with the reference genome, genome bedgraph of 139 genbank references for alignment comparison, and individual bed files of the seven patient samples with near-full length sequence, showing the contig mapping to the reference. Note that the assemblies appear artificially fragmented due to many dissimilar regions in the reference. (middle) shows the whole genome data clustered using the covariant allele density metric and UPGMA hierarchical clustering. (bottom) shows that same method restricted only to alleles used for multilocus sequence typing (MSLT) for *S. pneumoniae*. (left column) contains a tanglegram linking the two clustermaps and highlights the edges connecting the 7 SHAPU-derived samples (locations denoted in green). (right column) shows coordinate and region mappings moving vertically between panels. Samples are denoted at positions in clustering on far right.

All assembly breadth data calculated with BEDTools (Quinlan and Hall 2010) and the merged reference data was used to create the bedgraph visualization. Variant calling was completed using the alignments to call consensus variants after QC filtering. The vcf files were transformed into a level encoded matrix format and the covariant allele density was calculated as the count of the identical variants found in a pair of samples over the shared region covered by both samples, and the magnitude of this shared region (in basepairs) was used to normalize the metric. UPGMA hierarchical clustering was completed and the linkage topology compared between matrices generated from the set of all variants found in the samples (approximately whole genome comparison) to only those found in the

seven MLST genes (spi, recP, xpt, ddl, aroE, gdh, gki). The serotype metadata from the reference samples was added, along with the MLST types of the references and the cluster labels generated using the 'maxclust' criteria with parameter of 20. When cluster composition was compared directly using a maximum likelihood label mapping, the mean rand score was 0.903 indicating good agreement with cluster boundaries between linkages. Additionally, the tanglegram shows minimal intracluster crossings on well defined clades and has a low L1.5 norm entanglement score of 0.13. Combined with the observation that both the serotype and MLST label clustering generally observe the boundaries identified in the whole genome and MLST covariant allele density linkages, and the seven samples included are from a minimum of 4 separate major clades, this strongly supports the conclusion that there is no distinct strain type characteristic of SHAPU-derived *S. pneumoniae.*

Quinlan, A. R. and I. M. Hall (2010). BEDTools: a flexible suite of utilities for comparing genomic features. Bioinformatics **26**(6): 841-842.
